# Mandibular Mucormycosis: A Report of Four Cases and a Discussion on Their Management

**DOI:** 10.7759/cureus.30301

**Published:** 2022-10-14

**Authors:** Ejaz A Mokhtar, Naqoosh Haidry, Karishma ., Sumit Verma, Shahrukh Akbar

**Affiliations:** 1 Oral and Maxillofacial Surgery, All India Institute of Medical Sciences, Patna, Patna, IND; 2 Oral and Maxillofacial Radiology, All India Institute of Medical Sciences, Patna, Patna, IND; 3 Oral and Maxillofacial Surgery, B.R. Ambedkar Institute of Dental Sciences and Hospital, Patna, IND; 4 Oral and Maxillofacial Surgery, College of Dental Sciences, Bangalore, IND

**Keywords:** sars-cov-2, curettage, aggressive, conservative, mandibular mucormycosis

## Abstract

Mucormycosis of the mandible (MOM) is a rare fungal infection, and only 23 cases had been reported during the last 50 years worldwide, from seven different countries. Most of the cases were reported in India (n=8, 34%), followed by the United States (n=5, 22%). It is usually associated with an immunocompromised state and generally occurs after tooth extraction. Radiographically, it presents with the characteristic sign of osteomyelitis. Most of the previous case reports/series on MOM described successful outcomes with the resection of the involved segment. However, our experience in managing these cases was quite different and it was observed that resection is seldom required. It was seen that MOM rarely causes cortical perforation. One of the probable reasons is the thicker cortical bone and well-confined boundary of the mandible. Another reason could be the fulminating nature of the disease that leads it to rapidly spread in less resistant medullary bone before perforating cortical bone. During surgery, a clear line was seen separating necrotic medullary bone from healthy cortical bone. The thicker cortical bone of the mandible was found to be resistant to fungal invasion; however, the medullary bone was rapidly invaded. Therefore, the healthy cortical bone could be saved. The preservation of the cortical parts thus helps in maintaining the continuity of the bone. Surgical curettage of necrotic medullary bone is usually the optimal method to manage MOM affecting the mandibular body or ramus region.

## Introduction

The rarity of mucormycosis of the mandible (MOM) as compared to rhinocerebral mucormycosis could be attributed to different modes of entry into the human body [1]. The mode of entry of rhinocerebral mucormycosis is through the inhalation of fungal spores [2] from the environment where they are ubiquitously present. They get trapped in the paranasal sinus and due to immunocompromised conditions, they subsequently start colonizing. However, the fungal spores enter the mandible mostly through surgical wounds created normally by extraction sockets or, in rare cases, are hematogenous. In the immunocompromised state, the spores start colonizing in the medullary region and then spread in the cancellous region.

There has been a surge in rhinocerebral mucormycosis cases after the first and second waves of the coronavirus disease 2019 (COVID-19) pandemic, and it has been labeled post-COVID mucormycosis [3]. We also came across four cases of MOM.

Treatment guidelines exist for the management of mucormycosis involving the maxilla, sinus, orbit, and brain. Moreover, recently updated guidelines by the European Confederation of Medical Mycology (ECMM) and the International Society for Human and Animal Mycology (ISHAM) have addressed the management of rhinocerebral mucormycosis [4]; however, treatment guidelines are not available for the management of MOM. In this report, we present four cases of MOM and share our experience in managing them.

## Case presentation

Case series

A total of four cases including three males and one female were seen in our department from 2018 to 2021. The mean age of the patients at the time of diagnosis was 52.7.4 ±9.2 years. Two patients had bilateral involvement of the mandible while two patients had involvement of only the right side of the mandible. We managed all four cases. The modality of management and outcomes are summarized in Table 1. The modality of treatment was explained to the patients and informed consent was obtained from all of them. All cases were managed conservatively with curettage of medullary bone only. Here we discuss one of our cases in detail.

**Table 1:** Summary of four cases of mandibular mucormycosis* *Age of the patient, gender, location, predisposing factor, treatment performed, and outcome COVID-19: coronavirus disease 2019

Case no.	Age, years	Gender	Location	Predisposing factor	Treatment	Follow-up period, months	Outcome
1	47	Male	Right mandible body	COVID-19 infection	Debridement + amphotericin B + posaconazole tablet	16	Successful
2	46	Male	Bilateral mandible body + right angle and ramus of mandible	Diabetes	Debridement + amphotericin B + posaconazole tablet	15	Successful
3	52	Female	Bilateral mandible body + right angle and ramus of mandible	COVID-19 infection	Debridement + posaconazole tablet	10	Successful
4	66	Male	Right mandible body	COVID-19 infection + diabetes	Debridement + posaconazole tablet	9	Successful

Detailed report of one case and its management

**Figure 1:**
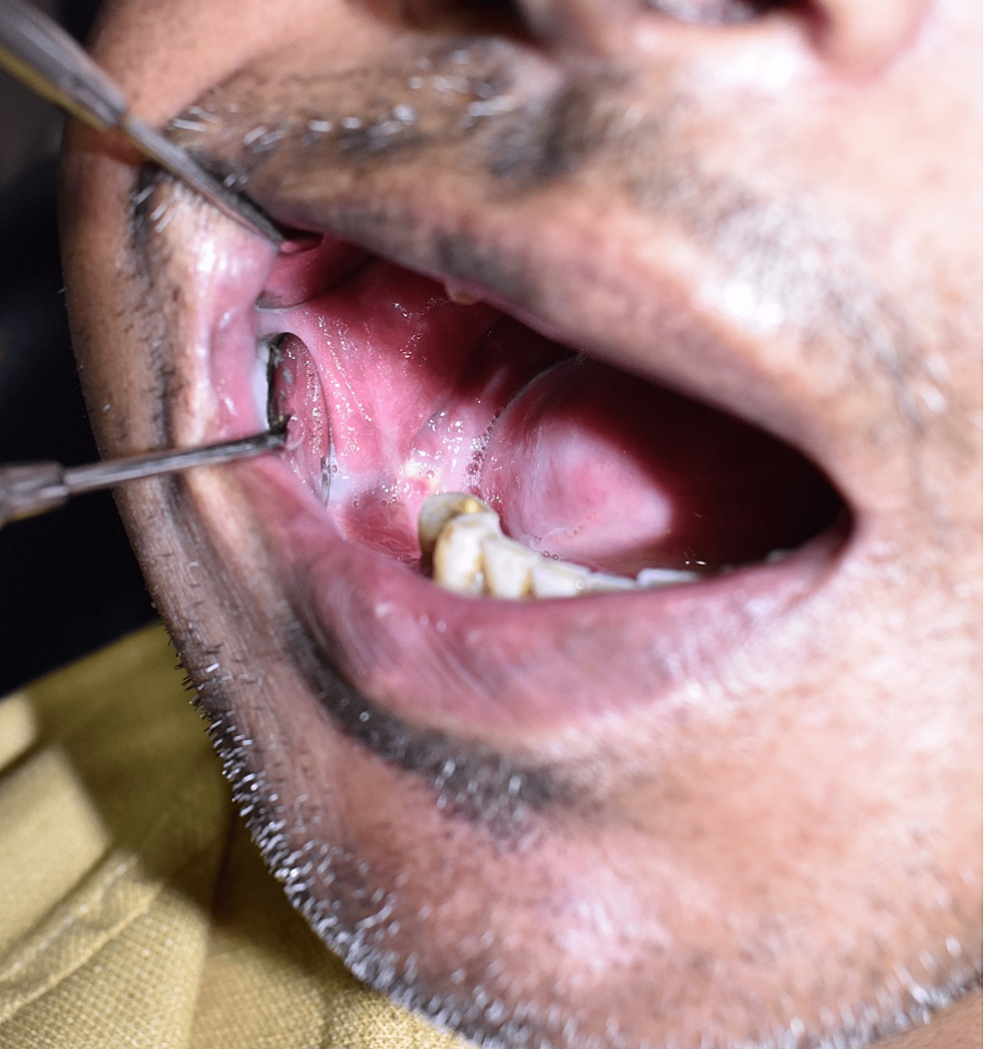
Intraoral picture showing extraction socket with pus discharge from the socket

**Figure 2:**
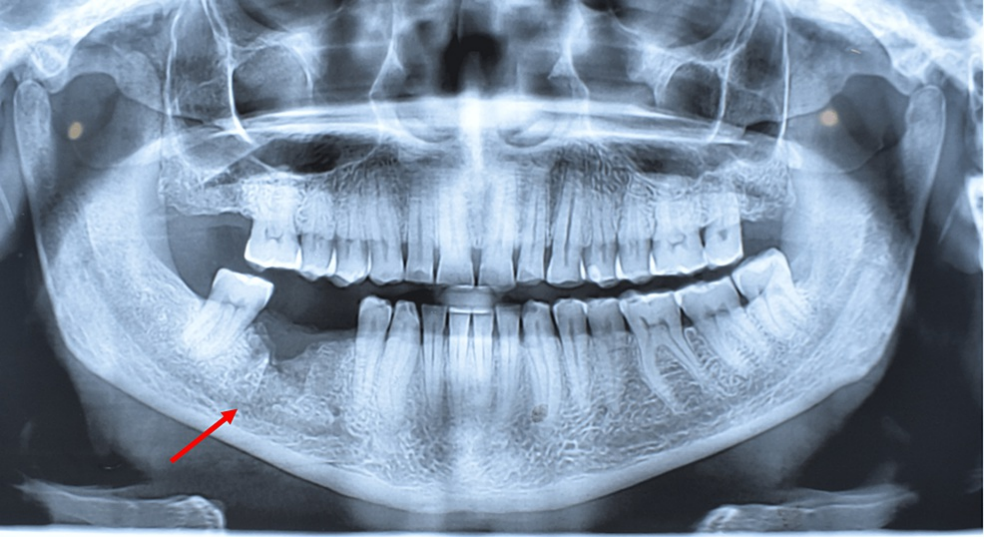
OPG showing extraction socket (arrow). No osteolytic lesion is seen OPG: orthopantomogram

**Figure 3:**
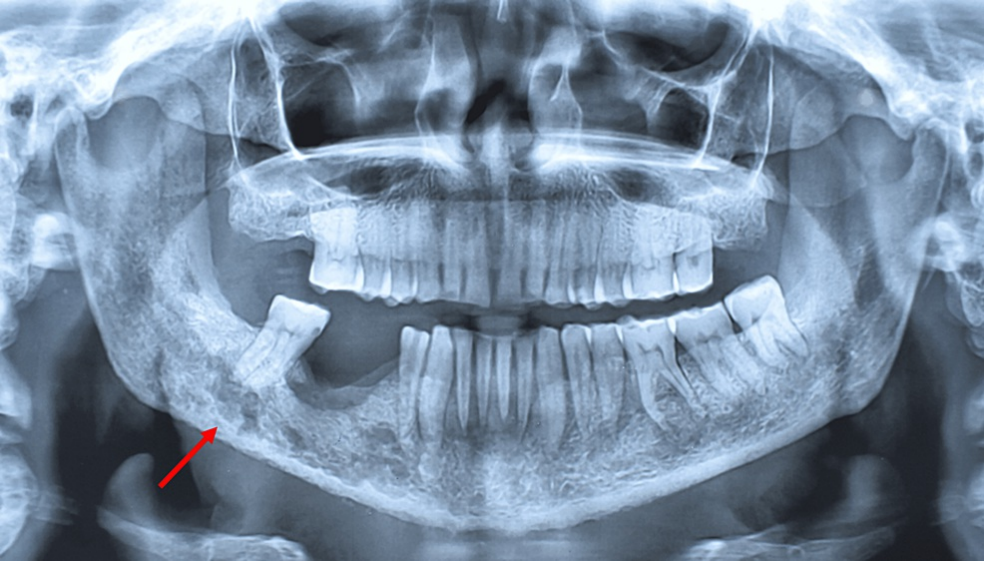
Repeat OPG one month after the first OPG showing osteolytic lesion (arrow) OPG: orthopantomogram

A 46-year-male patient with no previous history of COVID-19 presented with pain and swelling involving the submental and submandibular region with intraoral pus discharge from the right-side lower jaw for the last two months (Figure 1). He had a history of tooth extraction of the mandibular first molar of the right side three months prior. He had been suffering from diabetes for the last five years. The random blood sugar and Hb1Ac were 495 mg/dl and 15% respectively. An endocrinology consultation was taken and insulin (both Lantus and regular) administration was initiated to control elevated blood sugar levels. He had an orthopantomogram (OPG) done on December 12, 2020, which showed an extraction socket of the right-side lower first molar (Figure 2). The patient had a second OPG, which was taken after one month and showed an osteolytic lesion of the mandible involving the right ramus and body (Figure 3).

**Figure 4:**
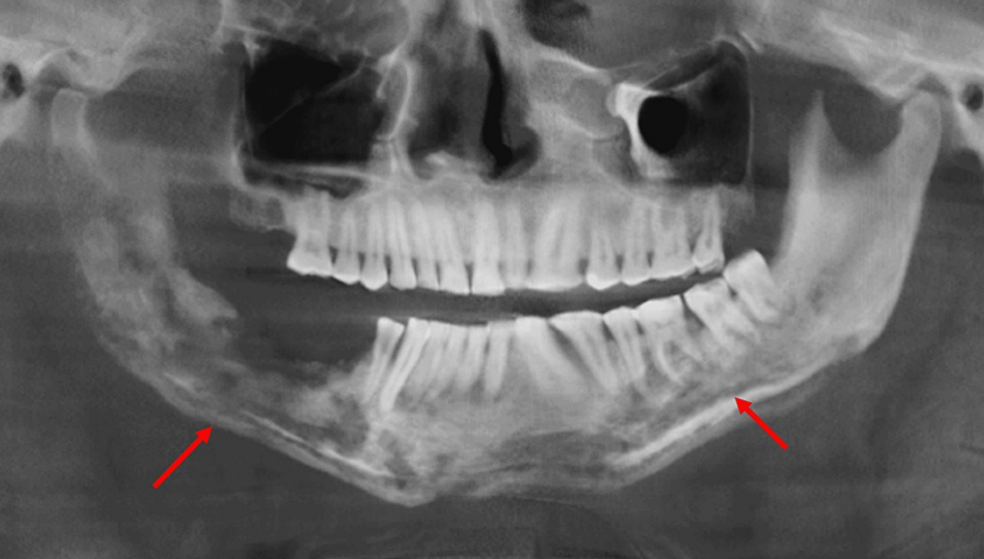
OPG reconstructed from the CBCT showing the progression of the osteolytic lesion to the left side (arrows) OPG: orthopantomogram; CBCT: cone-beam computed tomography

**Figure 5:**
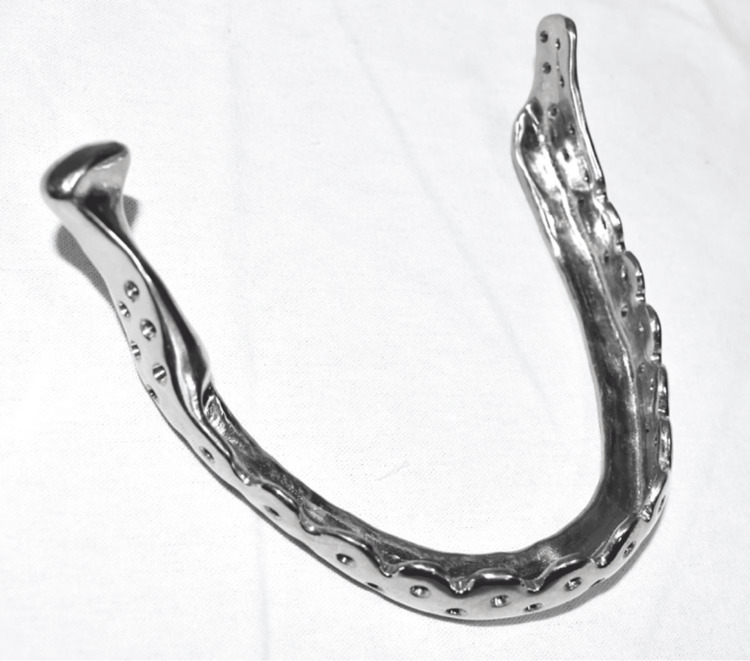
Patient-specific implant planned to be used for reconstruction after the resection of the mandible

Pus was aspirated from the right-side ramus region and sent for a GeneXpert test to rule out tuberculosis and for bacterial and fungal cultures. GeneXpert was negative for tuberculosis. Direct microscopy and culture were negative for fungal and bacterial elements. An incisional biopsy was sent for histopathology. In histopathology, aseptate hypha was seen, which confirmed mucormycosis. Cone-beam CT (CBCT) was again taken after 10 days while waiting for the histopathology report. The progression of the osteolytic lesion was seen on the left side of the mandible involving the left body and ramus (Figure 4). Liposomal amphotericin was then started with a daily dosage of 150 mg/day and a plan for the resection of the mandible was made from the left side of the condyle up to the right side of the ramus followed by reconstruction with a patient-specific implant (Figure 5).

**Figure 6:**
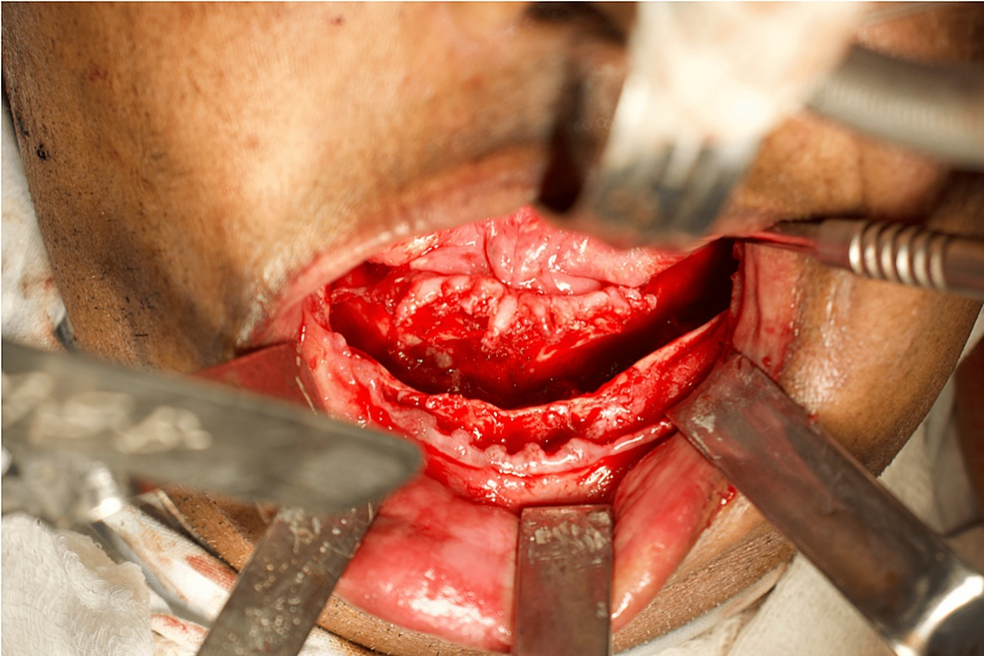
Intraoperative picture clearly showing intact bleeding cortical bone

**Figure 7:**
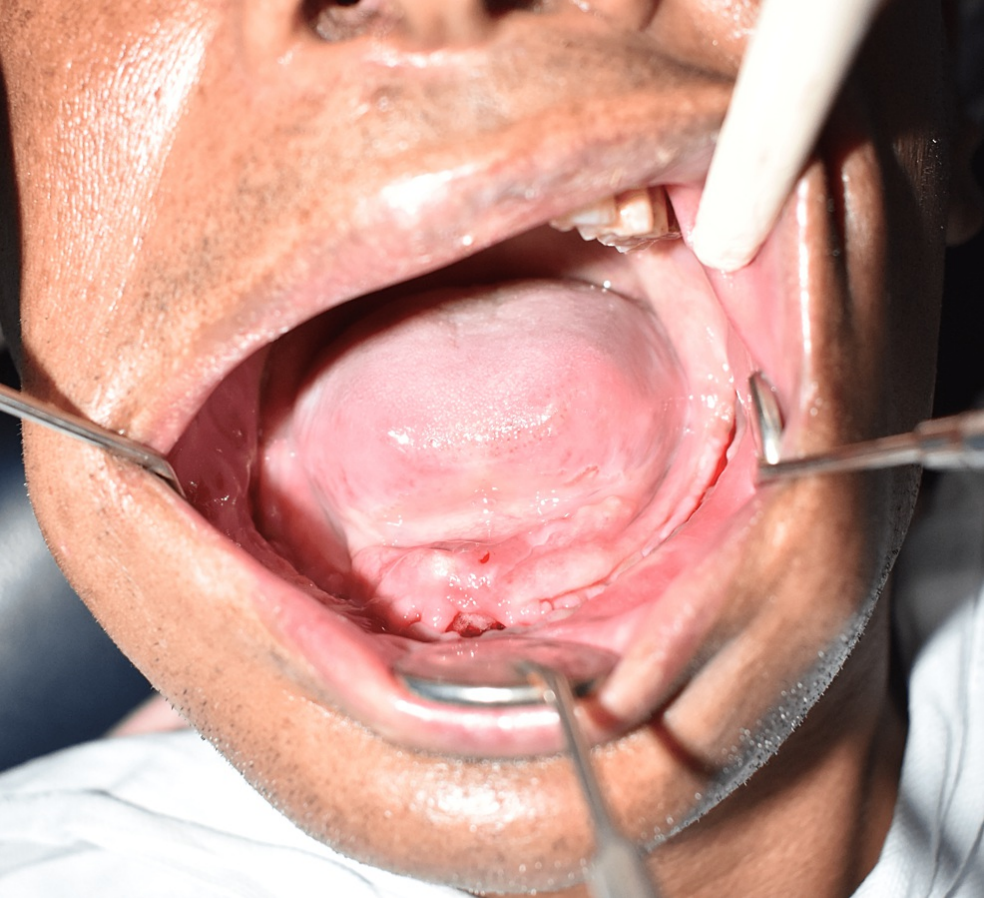
Postoperative intraoral photograph after two months of follow-up showing the healed surgical site

The patient was placed under general anesthesia. All teeth of the lower jaw were extracted. A crevicular incision was given on the buccal side and subperiosteal dissection was done to expose buccal and lingual cortices. Intraoperatively, buccal and lingual cortices were found intact. During surgery, a clear plane between necrotic medullary bone and bleeding buccal and lingual cortices were seen (Figure 6). Hence, the plan for the resection of the mandible was modified to debridement of necrotic bone and preservation of buccal and lingual cortices. Meticulous curettage of the osteolytic medullary region of the mandible was done, thereby removing all necrotic bone while both the cortices were saved. The wound bed was irrigated with the diluted hydrogen peroxide solution and the closure was primarily done. The wound got opened at some places in follow-up, which was managed with continuous irrigation with diluted hydrogen peroxide. After two months of follow-up, the wound was completely covered (Figure 7). A total dose of 2.9 gm of liposomal amphotericin B was given before switching to posaconazole tablet 100 mg three times a day with a loading dose of 600 mg. At the 10-month follow-up, the CBCT showed regeneration of the medullary bone. 

## Discussion

Mucormycosis is not an unfamiliar condition in India; the incidence rate before 2019 was almost 70 times higher than that in developed countries [5]. A 10-times surge has been seen globally in fungal infections with the emergence of the COVID-19 pandemic, and rhinocerebral and pulmonary mucormycosis has been the most common variant [6,7,8]. Corticosteroids, i.e., dexamethasone and methylprednisolone, are routinely prescribed for COVID-19 patients. Corticosteroids modulate inflammation-mediated lung injury and thereby reduce the progression of respiratory infection [9]. However, the side effects of corticosteroids, mainly increased secondary infections and the manifestation of latent diabetes, are the main causes of increased cases of mucormycosis in COVID-19 patients [10]. Diabetes was seen as the main predisposing risk factor either primarily or secondarily post-COVID-19 due to the indiscriminate use of steroids in 70% of cases of mucormycosis [11]. The hyperglycemic condition, especially ketoacidosis, provides an acidic environment that is suitable for the growth and multiplication of fungus. One of our four cases had a previous history of diabetes while three cases developed diabetes post-COVID infection due to steroid use.

MOM is usually caused by direct inoculation of spores to surgical sites or, in rare cases, is hematogenous, unlike rhinocerebral mucormycosis, which is transmitted through spore inhalation. All of our patients had a history of tooth extraction before developing mucormycosis. The fungal spore might have been inoculated at the extraction site.

Imaging plays an integral role in the diagnosis of osteomyelitis. Radiographically, it represents a loss of trabecular pattern of bone and osteolytic lesion involving the mandible. The extent of involvement seen in CBCT or CT may be less than the actual extent of the disease as reported previously in which pathology was seen concealing and the CT scan was unable to depict the actual extent of the disease [12]. The reason is the fulminating nature of the disease as it spreads rapidly in medullary space before causing cortical perforation.

Mucormycosis can be diagnosed by KOH mount, culture, or by histopathological examination. In our case series, three out of four cases were diagnosed on histopathological examination as direct smear examination was negative, while one case had been previously diagnosed. A high index of suspicion for MOM should be kept in mind when encountering patients with uncontrolled diabetes and osteolytic lesion involving the mandible.

Standard approaches for the management of coronavirus-associated mucormycosis are similar to the management of mucormycosis in non-COVID-19 patients, which are outlined in the ECMM-MSG global guidelines [4]. The treatment entails the reversal of the underlying condition, aggressive surgical management, and antifungal protocol. Decisions related to the treatment planning of isolated MOM can be challenging because there are no data from controlled studies that help to identify when conservative or more aggressive treatment strategies should be used. Among the 23 cases of MOM reported from seven different countries worldwide, surgical debridement or curettage was performed in 11 cases, in which only six (63%) patients survived while five (37%) patients died, mostly due to the progression of infection. Resection was performed on nine patients, of which eight cases survived while one patient died of cardiac arrest. Analyzing the success rate of resection over curettage, initially, the second resection of the involved area was planned; however, intraoperatively, after the removal of necrotic medullary bone, bleeding was noted from cortical plates, and hence the plan was changed to the preservation of bone as it was not clinically involved. Haidry et al. [8] have successfully managed a case of MOM, which had developed after COVID-19 infection, in a 59-year-old man by open surgical debridement. Similarly, Cohen et al. [13] have managed three cases, and Ambereen et al. [14] have managed one case of mandibular mucormycosis by debridement and curettage of the area.

Apart from the surgical part, medical management is also required in these patients. The mortality rate with medical treatment alone was 70% as compared to 14% in those treated both medically and surgically for rhinocerebral mucormycosis. Two of the patients were initially treated with liposomal amphotericin B and later on shifted to posaconazole. Case 1 and case 2 were given a cumulative dose of 3 gm, and 2 gm of liposomal amphotericin B before switching to posaconazole, while in the rest of the cases, only posaconazole was given. Posaconazole has been studied as salvage therapy in mucormycosis and is effective in 70% of cases of mucormycosis that were either resistant or allergic to amphotericin B [15]. It was found to be effective in all of our cases. Delayed-release posaconazole tablet is preferred over oral suspension because it displayed more reliable plasma concentration, less interaction with other drugs, and no added side effects, and it is cheaper than amphotericin B. Prophylaxis with parconazole tablet is initiated with a loading dose of 300 mg twice daily on the first day and then continued at a dose of 300 mg once daily [16,17].

## Conclusions

MOM could be managed successfully with curettage of the medullary bone alone. Fungal hyphae can rapidly invade the medullary bone; however, the cortical bone seems resistant to invasion by fungi. Hence, the cortical part of the mandible could be saved, which will maintain the continuity of the bone. A clear cleavage plane was seen between the medullary necrotic bone and bleeding cortical bone. Hence, the cortex may be preserved, which will decrease the morbidity associated with the surgery.
